# Supplementary figures and images for: SLC35A2 gene product modulates paramyxovirus fusion events during infection
Source: PLoS Pathog. 2025 Jan 10;21(1):e1012531. doi: 10.1371/journal.ppat.1012531 (PMC11756793; doi:10.1371/journal.ppat.1012531)

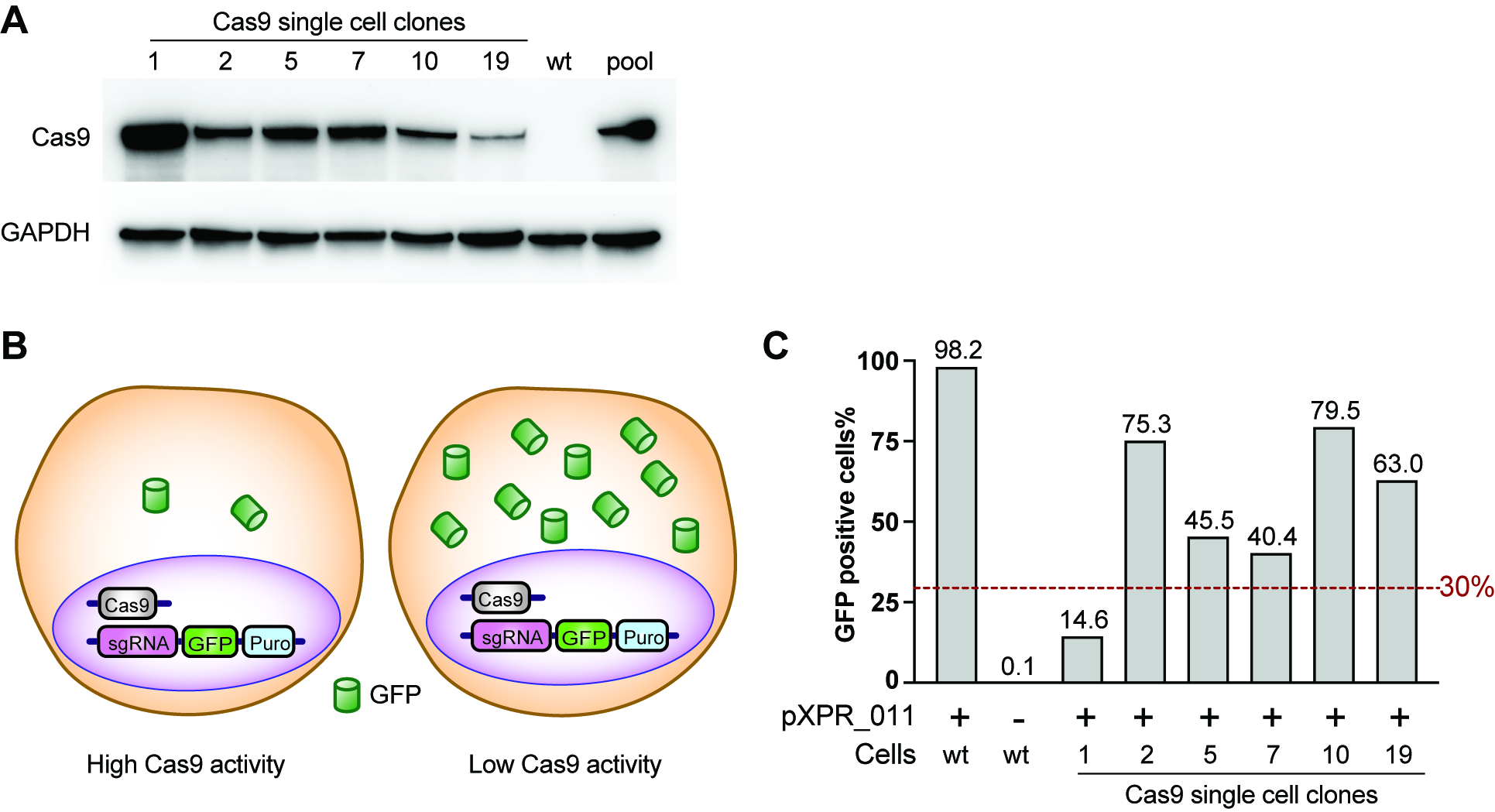

Supplement: S1 Fig — (A) The Cas9 expression of 6 A549-Cas9 single cell clones, A549wt cells, and A549-Cas9 pool was detected by western blot. GAPDH expression was determined as loading control. (B) Diagram of Cas9 activity assay: with Cas9, GFP, and sgRNA targeting GFP in the same cells, higher Cas9 activity leads to lower GFP intensity, and lower Cas9 activity leads to higher GFP intensity. (C) Percentage of GFP-positive cells detected by flow cytometry after transduction of the pXPR_011 plasmid into A549 wt cells and A549-Cas9 single cell clones. A549 wt cells without transduction were used as a negative control. The red dashed line indicates 30% of GFP positive cells; it is generally accepted that Cas9 cells with less than 30% GFP-positive cells can be used for CRISPR screening. (TIF) [file ppat.1012531.s001.tif]

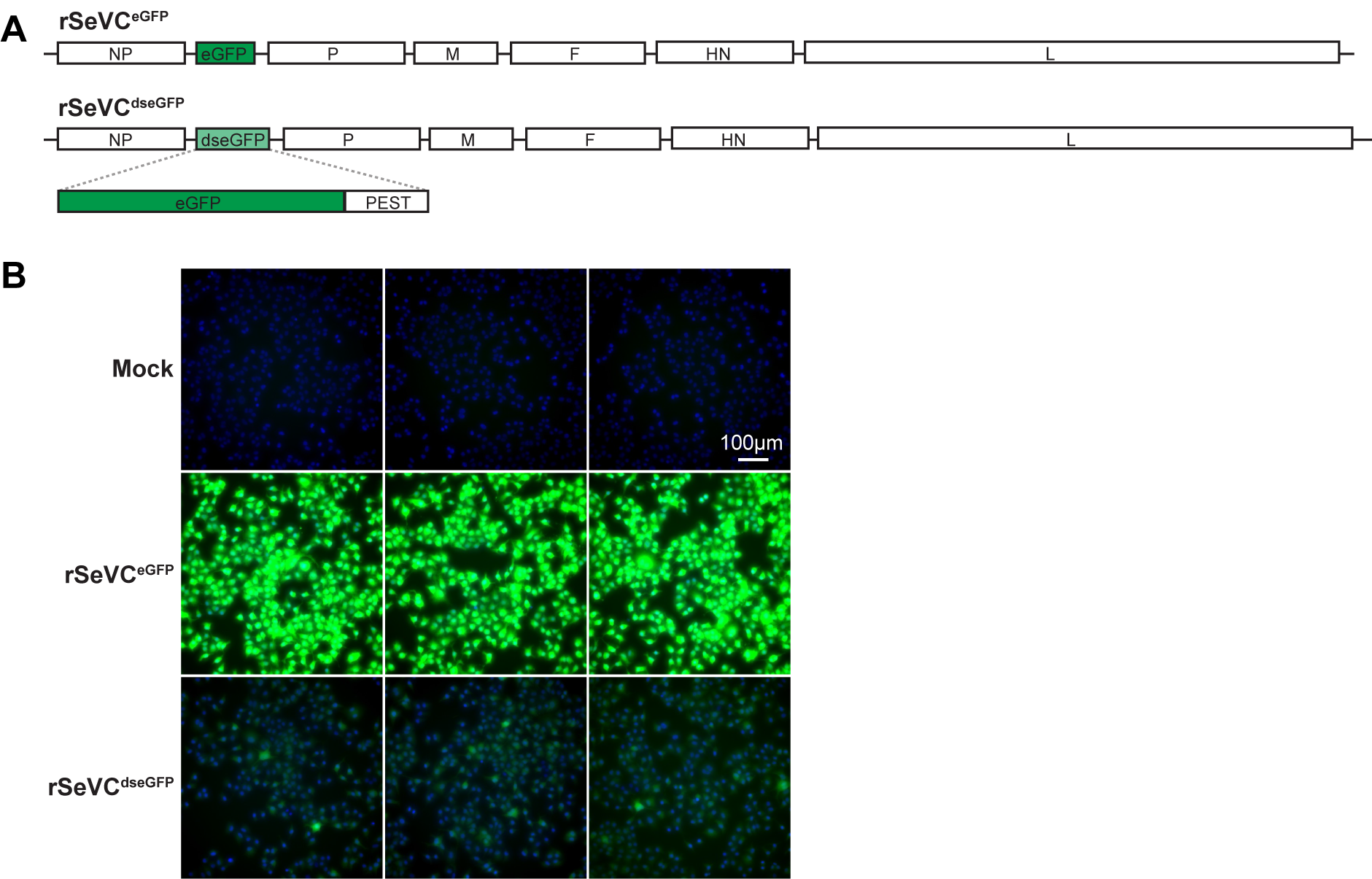

Supplement: S2 Fig — (A) Schematic representation of the SeV reporter viruses. eGFP or dseGFFP gene was inserted into the SeV genome between NP and P gene. dseGFP is made by fusion of a PEST degradation sequence to the C terminal of eGFP. (B) Fluorescence images of A549wt cells infected with rSeVeGFP and rSeVdseGFP. A549wt cells were infected with an MOI of 3 of rSeVeGFP or rSeVdseGFP and images were analyzed at 24hpi. The nucleus was stained with Hoechst 33342 (Blue), green fluorescence indicates viral infection eGFP or dseGFP expression and accumulation. The images display three different fields of view. Scale bar lengths are indicated. (TIF) [file ppat.1012531.s002.tif]

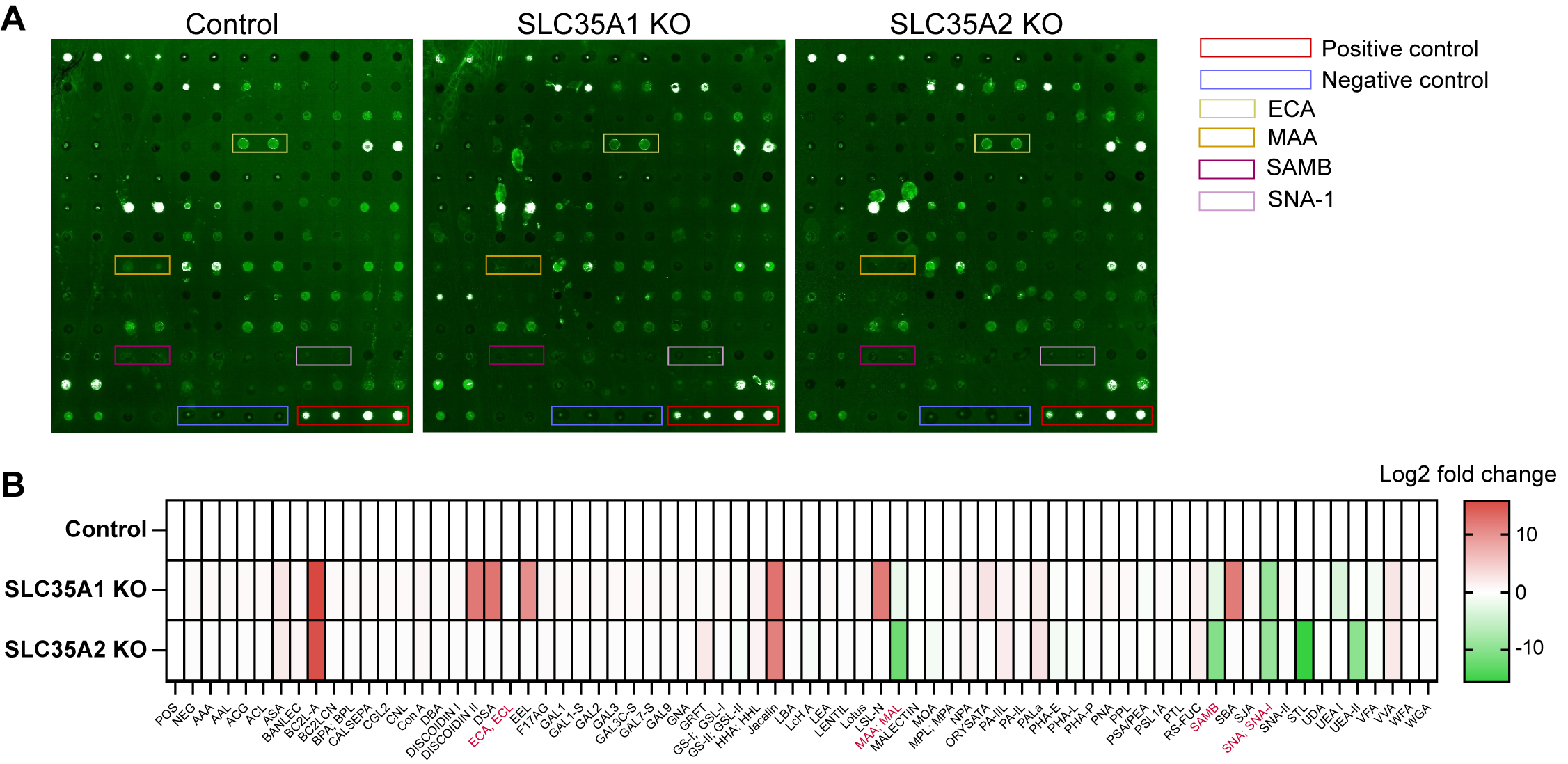

Supplement: S3 Fig — (A) Lectin array images of control and KO cell lines. Cell lysates were analyzed using a Lectin-70 array, with selected sialic acid- or galactose-binding lectins highlighted. (B) Log2 fold change heat map. A heat map showing lectin array intensities for the three different cell lines. Fluorescence intensities were extracted from the lectin arrays using the Gal.file. Data for SLC35A1 KO and SLC35A2 KO cells were normalized to the control using positive control wells in each array. Log2 fold change was calculated based on normalized fluorescence intensities relative to control cells. Red indicates increased binding, while green indicates decreased binding. (TIF) [file ppat.1012531.s003.tif]

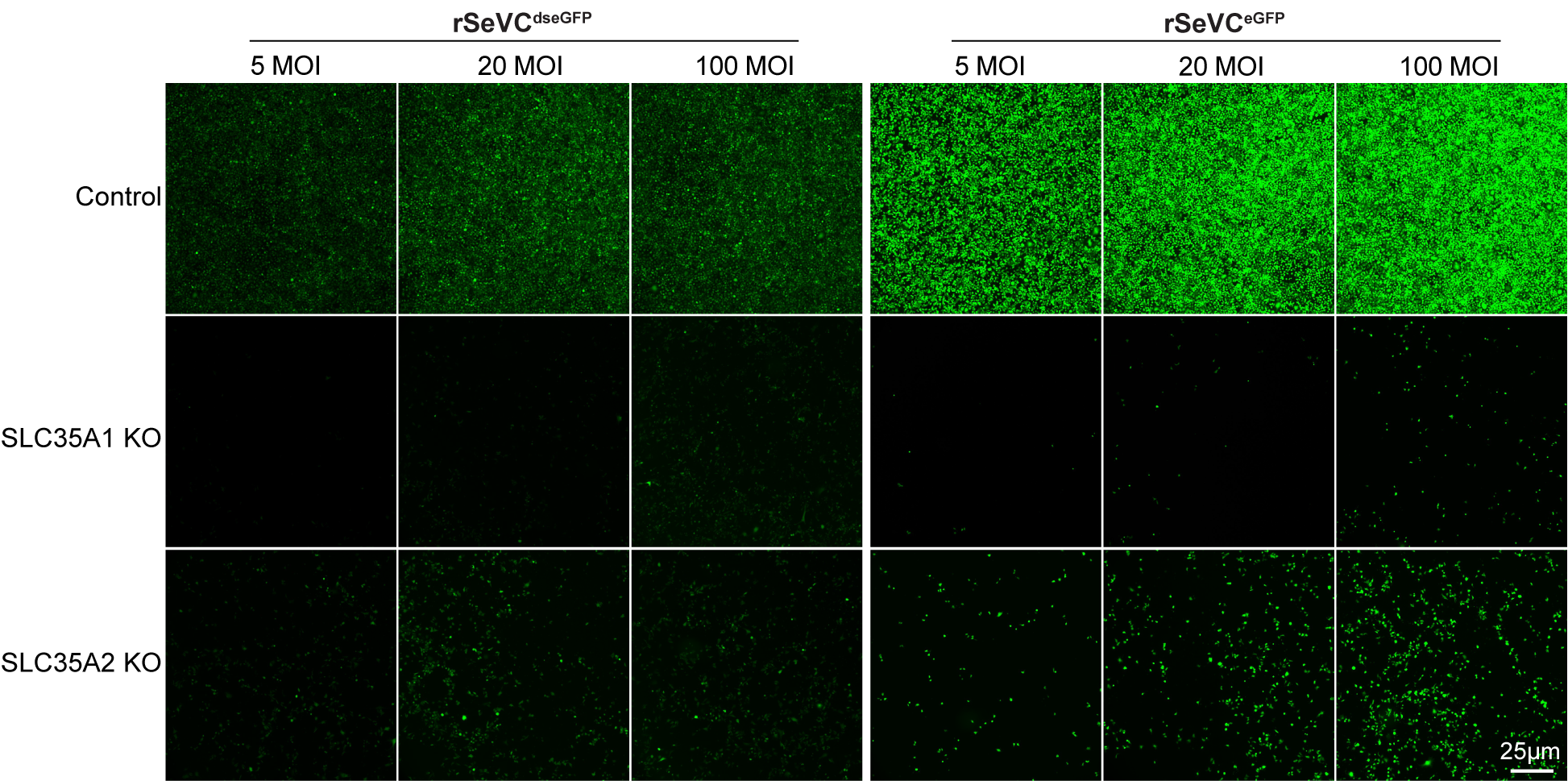

Supplement: S4 Fig — Fluorescence images showing GFP expression in control, SLC35A1 KO, and SLC35A2 KO cells infected with rSeVCeGFP or rSeVCdseGFP at MOIs of 5, 20, or 100, 24hpi. Scale bar lengths are indicated. (TIF) [file ppat.1012531.s004.tif]

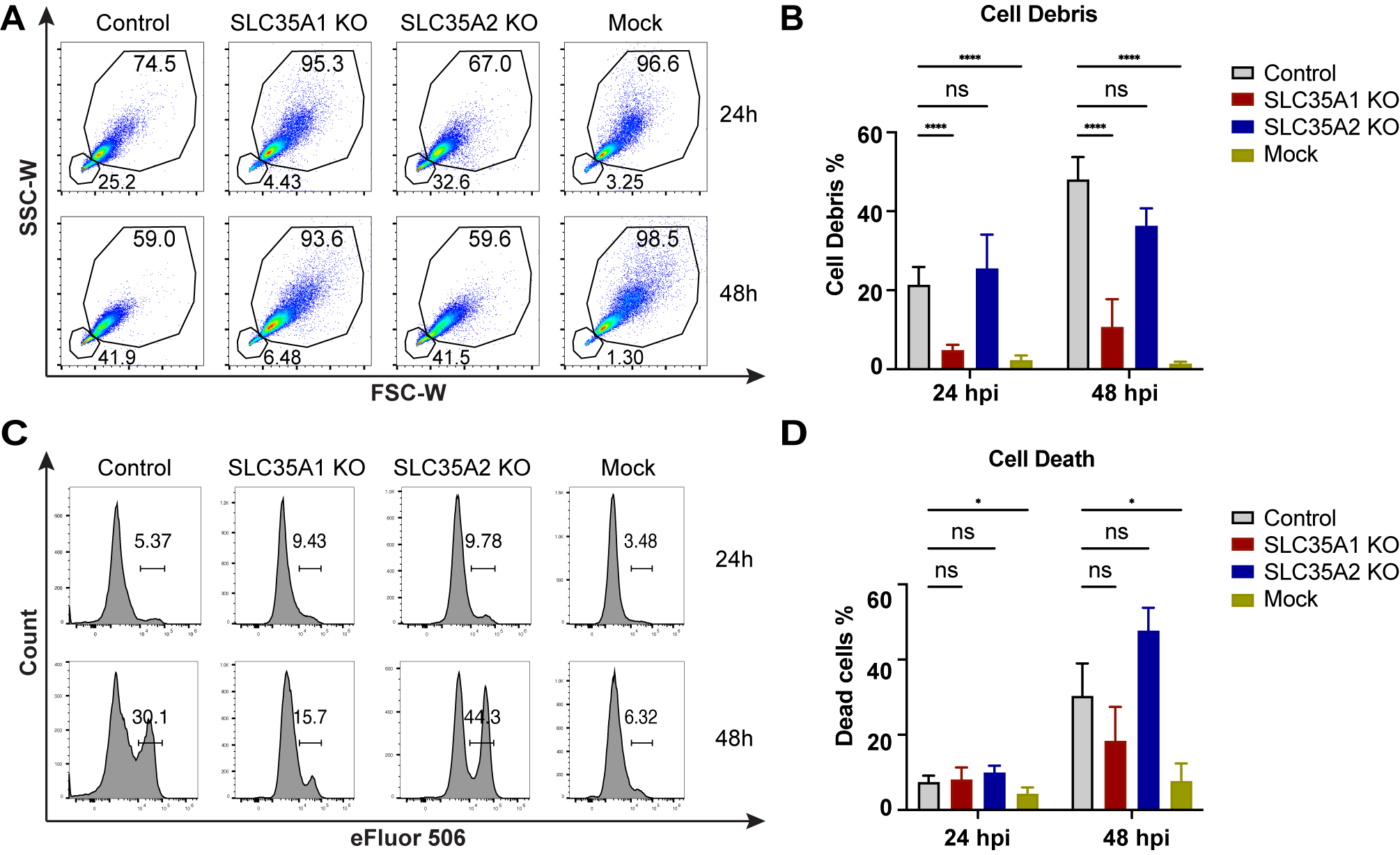

Supplement: S5 Fig — (A) Flow cytometry analysis of cell debris and cells in control, SLC35A1 KO and SLC35A2 KO cells infected with rNDVeGFP at an MOI of 1.5 and control mock cells at 24 and 48 hpi. The percentages of cell debris or cells are indicated within each plot. Data shown represent one of three independent experiments. (B) Quantification of cell debris percentages at 24 and 48 hpi. Data represent the mean of three independent experiments. Statistical significance is indicated as follows: ****p < 0.0001, ns = not significant. (C) Flow cytometry analysis of cell death of cells population from (A) in control, SLC35A1 KO, SLC35A2 KO, and mock-infected cells at 24 and 48 hpi, as indicated by Fixable Viability Dye eFluor 506 staining. Percentages of dead cells are indicated within each plot. Data shown represent one of three independent experiments. (D) Quantification of dead cells percentages at 24 and 48 hpi. Data represent the mean of three independent experiments. Statistical significance is indicated as follows: *p < 0.05, ns = not significant. (TIF) [file ppat.1012531.s005.tif]

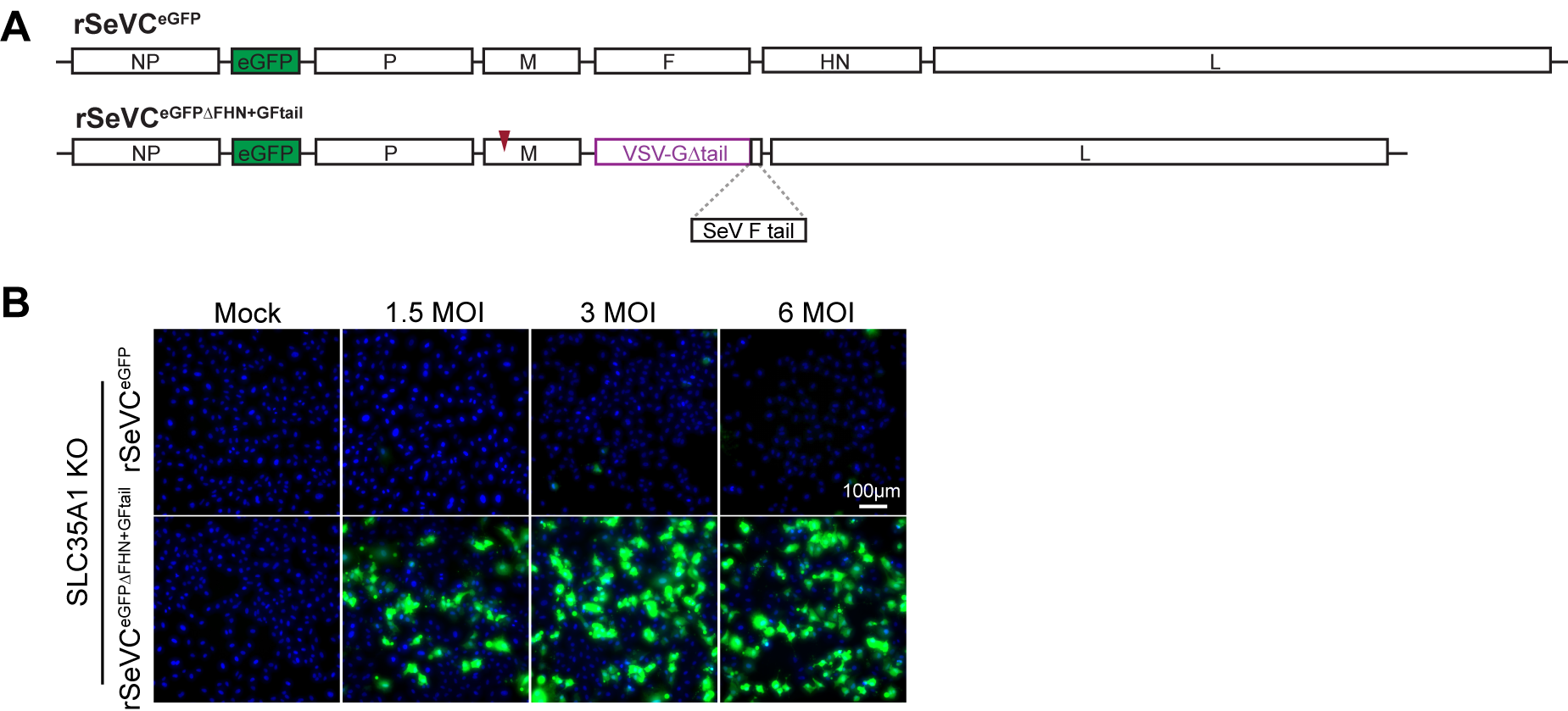

Supplement: S6 Fig — (A) Schematic representation of the rSeVCeGFPΔFHN+GFtail. rSeVCeGFPΔFHN+GFtail was designed by replacing SeV F and HN gene with a GSV-G deleting its tail and fusing with SeV F tail on rSeVCeGFP. Red inverted triangle indicated a mutation on M protein. (B) Fluorescence microscopy images of SLC35A1 KO cells infected with rSeVCeGFPΔFHN+GFtail at MOIs of 1.5, 3, and 6. Images were analyzed at 24 hpi. The nucleus was stained with Hoechst 33342 (blue), and green fluorescence indicates viral infection, as shown by eGFP expression. rSeVCeGFP and Mock-infected cells were used as controls. Scale bar lengths are indicated. (TIF) [file ppat.1012531.s006.tif]
