# Supplementary material for: SLC35A2 gene product modulates paramyxovirus fusion events during infection
Source: PLoS Pathog. 2025 Jan 10;21(1):e1012531. doi: 10.1371/journal.ppat.1012531 (PMC11756793; doi:10.1371/journal.ppat.1012531)
Supplement: Attachment — Submitted filename: Point by Point Response .docx [file ppat.1012531.s011.docx]

**Point by Point Response**

We thank the reviewers for their constructive revision to our manuscript. We have addressed all the concerns that have resulted in a much-improved report. All revisions are marked in burgundy font in the “marked” copy of the manuscript. Below a point-by-point response to all your concerns:

**Reviewer #1 (Remarks to the Authors):**

The paper by Yang et al makes a nice contribution to understanding host molecules that affect paramyxovirus entry/fusion.

The set of experiments that is described here first detail a loss of function screen for host factors in paramyxovirus infection and identify two transporters involved in adding carbohydrates (ultimately, sialic acids) to glycoproteins and glycolipids, encoded by SLC35A1 and SLC35A2. Both are essential for infection by sendai virus (a model paramyxovirus). Host cell sialic acid processing (and expression on cell surfaces) has been known to be critical for paramyxovirus entry, and these findings make sense in the context of what is known. The paper then proceeds to assess the distinction between the roles of these two transporters, and the effects of KO of these transporters, on a series of different paramyxoviruses (NDV and mumps in addition to Sendai). The findings are interesting in that they extend the notion of the differences in receptor density requirements in different infection conditions and between viruses; the differences in receptor density requirements for viral entry vs. viral spread; and the comparison of these properties among sialic acid-binding paramyxoviruses.

The experiments raise interesting questions that will merit further investigation. A number of mechanistic questions remain open. I have a few questions below that the authors may wish to address.

1. There is an intersecting set of factors that affect the outcome of infection in this paper, and the roles of (a) cell surface sialylated receptor type; (b) cell surface receptor density; (c) cell type are entangled as to cause/effect. I would like this to be somewhat clearer.

We thank the reviewer for all their thoughtful suggestions. We have now expanded the manuscript discussion to acknowledge the contribution of these factors to infection in the context of our results.

2. The confirmation that sialic acid moieties on the surface are essential host factors may be more firmly placed in context of earlier discoveries. This portion of the paper could be shortened significantly so that the paper can focus on the more novel parts.

Although the role of SA as a receptor for paramyxoviruses is well accepted, we could not find any formal demonstration that its transporter SLC35A1 is directed implicated in paramyxovirus infection. Because of this, we’ve decided to keep most of this section.

3. The impact of the KO experiments directly depends on the specific cell lines used in each experiment, as entry/fusion is highly dependent on the specific cell and composition of the cell membrane. The systems used here are highly lab adapted – and while it’s totally justifiable to use model systems, the comparisons are valid only in relative terms and if the comparisons are made of infections in the same cell lines. This should be clarified so that it’s evident which factors are being held constant.

To clarify, we used A549 cells in all experiments. We revised the text to more explicitly say this in all figure legends and relevant results sections. In addition, we have expanded the discussion including a section on the impact of cell types in the interpretation of our data.

4. Just as the infection is affected by the KO of one or the other sia transporter, the cell line being used and the virus strain being used also impacts receptor usage and the ratio of entry to spread (etc) and these factors (cell line, virus strain) should be carefully discussed.

We have expanded the manuscript discussion to include these factors.

5. In terms of receptor usage (e.g.lines 196-9) it seems that the authors are implying but not directly saying that there is a receptor usage difference between the paramyxoviruses that are tested. This should be clearly stated with the references to receptors differentially used and their sia status (if known) to make sense of these findings.

This statement has been rephased to (now in lines 223-227): “Interestingly, for both NDV and MuV infections, but no for SeV, more infected cells were observed in SLC35A2 KO cells than in double KO cells (Fig. 4B and 4C), suggesting that *SLC35A2* and *SLC35A1* have non-redundant functions during paramyxovirus infection and that *SLC35A2* differentially impacts infection with different paramyxoviruses”.

In addition, we have included the following in the discussion (lines 403-412): “The paramyxoviruses used in this study failed to infect *SLC35A1* KO cells due to the lack of cell surface sialic acid receptors. Interestingly, although SeV failed to infect *SLC35A2* KO, it attached to these cells but with less efficiently than to control cell. The ability of SeV to attach to cells lacking galactose suggests that it utilizes alternative sialylated surface receptors beyond the classic sialic acid linked to galactose previously reported [4, 12, 13, 48-54]. Alternative linkages, such as α2,6-linkage to N-acetylgalactosamine have been reported [4, 55]. SeV and MuV are reported to only use α2,3-linked sialic acid to attach to cells [12-15], while NDV can bind both α2,3-linked and α2,6-linked sialic acids [16]. Based on the data reported here, we hypothesize that either α2,6-linkage to N-acetylgalactosamine or other sialic acid linked molecules contribute to SeV entry into A549 cells.”

6. The sections on differential infection vs spread (e.g.lines 213-222) are very interesting and merit development. There have been various thoughts for paramyxoviruses about the different requirements for entry vs. spread, and publications that relate to this question. As above, this will differ greatly (entry vs. spread for each of these viruses) depending on the cell line used (and the specific strain). This topic in the paper needs clarity and would benefit from some additional development and discussion.

Our interpretation on the different requirements between infection and spread are directly related to the potential differential mechanisms involved in virus-cell and cell-cell fusion. We have expanded our discussion on these topics by adding: “This finding suggests that *SLC35A2* promotes MuV cell-to-cell transmission through MuV induced syncytia formation. *SLC35A2* KO doesn’t affect MuV-cell fusion but decreased MuV induced cell-cell fusion, suggesting there are differences between the mechanisms of virus-cell fusion and cell-cell fusion. The ability of viruses to trigger cell-cell fusion between infected cells and surrounding non-infected target cells is mainly related to virus-encoded fusion proteins expressed at the cell surface of the infected cells and cell-cell fusion is then mediated by interactions of viral fusion proteins with surface molecules or receptors expressed on neighboring non-infected cells [64]…”

7. Regarding this topic for mumps infection (e.g. lines 290-299) it would be of interest to know here, what do the differential responses to entry/infection vs. syncytia production say about receptor usage by these viruses? And, are the syncytia biologically meaningful or are they are tissue culture artifact – or a surrogate (as the authors imply) for a persistent mode of infection? These questions should be clarified.

We have including this in the discussion shown above.

8. The work contains many fascinating observations and offers interesting observations about these processes for several lab strain paramyxoviruses in cell lines. It would benefit the paper if the conclusions that may be drawn and the lines of evidence could be cleanly summarized and the points above about the influence of cell line vs viral receptor usage could be integrated into those conclusions. Currently the information is somewhat diffusely presented and some of the potentially very interesting aspects of these comparisons are not summarized. Regardless of the artifactual nature of the systems, if the viruses / pathways are placed on a level field, the comparisons are potentially very interesting.

We thank again the reviewers for thoughtful contributions to our manuscript. We believe we have significantly clarified all these points as explained above.

**Reviewer #2 (Remarks to the Authors):**

The authors have used a loss-of-function CRSPR screen to identify host factors critical for paramyxovirus infection. They used Sendai virus for the initial screen in A549 cells, where they identified CMP-sialic acid transporter (CST) gene SLC35A1 and the UDP-galactose transporter (UGT) gene SLC35A2 as essential for Sendai virus infection. They then proceeded to validate these hits in mumps virus and Newcastle disease virus. They made single and double knockout cells and proceeded to show that SLC35A1 was essential for virus binding, as knocking it out eliminated cell surface sialic acid, the receptor for the viruses tested. The importance of SLC35A1 in influenza virus and porcine delta coronavirus binding and entry have already been reported. On the other hand, SLC35A2 seemed to be involved in virus-cell fusion in the case of Sendai virus and cell-cell fusion in the case of mumps virus. This is the first report on the possible role of SLC35A2 in membrane fusion and as such is an intriguing finding that warrants further study.

Major: 
Line 243 and Fig 6A: The authors state that SeV binding to SLC35A2 KO cells was lower than that of control cells. It may be helpful to the reader if this was quantified, perhaps as mean fluorescence intensity (MFI).

We thank the reviewer for this comment and agree that quantifying SeV binding using MFI is helpful. The quantification is now shown in Figure 6B.

Minor: 
1. Line 56: Italicize SLC35A1.

Corrected.

2. Line 71-74: While the authors state that they used a rSeV that allowed for more sensitive and accurate analysis of CRISPR-Cas9 knockout, it is not clear how this is achieved using this construct. Part of the answer is revealed in the Discussion (lines 368-370). The authors should consider moving this explanation to the Introduction section where the rSeV is first described or to the Results section when the authors describe the use if this rSeV in the CRSIPR screen.

The following paragraph is included in the results section: “For screening, we generated a recombinant SeV expressing a destabilized eGFP (rSeVCdseGFP). This virus was generated by inserting a destabilized eGFP (dseGFP) between SeV NP and P genes (Fig S2A). **Destabilization of the protein is achieved by fusing a proline-glutamate-serine-threonine-rich (PEST) peptide to eGFP. This peptide reduces the half-life of GFP from 20 hours to 2 hours causing a 90% signal loss [36, 37].** As shown in Fig S2B, the rSeVC^dseGFP^ did not show signs of attenuation in virus titer (10^8.28^ vs 10^8.35^ TCID_50_/ml) but exhibited lower eGFP intensity compared with rSeVC^eGFP^ in infected A549 cells.”

3. Fig 1B and 1D are not called out in the main text.

Thanks for catching this oversight. We have now corrected it.

4. Line 164: rSeVCmiRF670 was abruptly introduced without an explanation of what this construct is and why it was used.

We have now explained what rSeVC^miRF670^ is and why it was used in lines 190-193: “Since the complemented *SLC35A1* and *SLC35A2* are fused with GFP, in these experiments we used a SeV miRF670 reporter virus rSeVC^miRF670^ in which a monomeric near-infrared fluorescent protein (miRFP670) was inserted into the SeV genome between the NP and P genes.”

5. Line 190: “look” should be “looked”.

Corrected.

6. Line 194-196: This statement is difficult to follow. Please rephrase to help the reader.

The statement was rephrased to (now in lines 223-227): “Interestingly, for both NDV and MuV infections, but no for SeV, more infected cells were observed in SLC35A2 KO cells than in double KO cells (Fig. 4B and 4C), suggesting that *SLC35A2* and *SLC35A1* have non-redundant functions during paramyxovirus infection and that *SLC35A2* differentially impacts infection with different paramyxoviruses”.

7. Line 257: “affects” should be “affect”.

Corrected.

8. Line 267-8 and Line 319: These statements should be qualified to indicate that SLC35A2 does increase the efficiency of SeV binding to cells.

Thanks for pointing this out, we agree that it should be clearer. We have now clarified that SLC35A2 is not essential for SeV binding but can enhance binding efficiency, as mentioned in lines 287 and 392.

9. Line 313: “transporters” should be “transporter”.

Corrected.

10. Line 326: delete “a”.

Corrected.

11. The authors use both “dsGFP” and “dseGFP” in the text. Are these the same construct or different?

Thanks for pointing this out. “dsGFP” should be “dseGFP”. We have now changed all “dsGFP” to “dseGFP”.

12. Discussion of how SLC35A2 may impact membrane fusion was lacking.

We agree that this was an important missing discussion. We have now added the following statement to the discussion (lines 423-426 and 443-456):

“In this study, the virus stocks used to infect cells were infectious, even when MuV particles were produced in *SLC35A2* KO cells, suggesting that during paramyxovirus infection virus-cell fusion and cell-cell fusion event are influenced by changes in host cells. The specific molecular mechanisms involved require further investigation”.

“This finding suggests that *SLC35A2* promotes MuV cell-to-cell transmission through MuV induced syncytia formation. Interestingly, *SLC35A2* deficiency didn’t affect MuV-cell fusion but decreased MuV induced cell-cell fusion, suggesting differences between the mechanisms of virus-cell fusion and cell-cell fusion. The ability of viruses to trigger cell-cell fusion between infected cells and surrounding non-infected target cells is mainly related to the expression of the viral F at the surface of the infected cells and cell-cell fusion is then mediated by interactions of viral F with surface molecules or receptors expressed on neighboring non-infected cells. A two-component system, in which the first component consists of transmembrane receptors and the second component consists of the multiprotein fusion complexes, could be used to mediate cell-cell fusion events [64] and cell surface fusion proteins such as fertilin and meltrin α may involve in this process [65]. *SLC35A2* KO may influence the glycosylation of host glycoproteins required for SeV-cell fusion and MuV-induced cell-cell fusion, but not for MuV-cell fusion. Therefore, further investigation into the specific host-side mechanisms is needed”.

**Reviewer #3 (Remarks to the Authors):**

Yang et al. found that sugar nucleotide transporters, SLC35A1 and SLC35A2, were critical factors facilitating various paramyxoviruses through the genome-wide CRISPR KO. The authors mentioned that SLC35A2, the UDP-galactose transporter, played some roles in the viral fusion step. The observation was potentially interesting; however, some of the descriptions were not fully supported by the experimental results.

Major:

1. Through the manuscript, the effect of SLC35A2 on the fusion activity of SeV was evidenced via the results of Fig. 3B. However, the settings of 400 MOI were unlikely in routine experiments. Since this experiment provided very important evidence for the core statement of this manuscript, the result should be confirmed by multiple experimental methods.

Based on the context of the comment, we believe that the reviewer refers to Fig 6B which is now Fig. 7B. The extremely high MOI is standard for binding, fusion, and internalization assays given the short time needed to detect rare events. (Some examples can be seen in ref [1-3].

In addition, to confirm our SeV-cell fusion results, we have now added experiments to directly visualize the virus-cell fusion using electron microscopy. The new data is shown in Fig.7C-F.

2. The author evaluated cellular glycans via staining with lectins. However, KO of a certain glycan-synthesis machinery often resulted in unexpected changes in global cellular glycome. I highly encourage the authors to evaluate the cellular glycans using glycomic approaches such as MS or lectin array.

Thanks for the suggestion, we have included lectin array data to show global cellular glycans changes which are shown in new supplementary Figure 3. We have also added a discussion on these data how it may explain changes in fusion events. The discussion can be found in lines 426-437.

3. I could not find a detailed description of miRF670.

We have now added an explanation of what rSeVC^miRF670^ is and why it was used in lines 191-193.

4. This is an optional comment. To clarify the role of SLC35A2 on viral infectivity, propagating the virions in SLC35A2-KO cells and checking the infectivity of the virions in other permissive cells perhaps make sense. If the glycosylations of the virions critically contribute to the loss of function, the fusion process would be disabled in the virions propagated in the KO cells. In the other case, cellular SLC35A2 would support the fusion process in some way.

Since SeV does not fuse with SLC35A2 KO cells we are unable to get SeV virions generated from SLC35A2-KO cells. For MuV, as we show in Figure 8A and B, we used SLC35A2-KO cells to propagate virions with 1.5 or 15 MOI and measured TCID50 in permissive A549 cells. As shown in Figure 8A, SLC35A2 KO cells can produce the same level of infectious viral particles as control cells. These data indicate that SLC35A2 plays a role in MuV-induced cell-cell fusion but doesn’t affect MuV’s infectivity.

Minor:

The experiment in Fig. 3B is hard to understand. An illustration of the principle of the experiment will help the readers easily understand.

Based on the context, we believe that the reviewer you are referring to Fig 6B which is now Fig. 7B. We have now added an illustration shown as new Fig.7A to facilitate understanding of the experimental design.

**References:**

[1] Carette, J. E., Raaben, M., Wong, A. C., Herbert, A. S., Obernosterer, G., Mulherkar, N., ... & Brummelkamp, T. R. (2011). Ebola virus entry requires the cholesterol transporter Niemann–Pick C1. Nature, 477(7364), 340-343.

**An MOI of 200-500 was used for a fusion assay.**

[2] Rosmarin, D. M., Carette, J. E., Olive, A. J., Starnbach, M. N., Brummelkamp, T. R., & Ploegh, H. L. (2012). Attachment of Chlamydia trachomatis L2 to host cells requires sulfation. Proceedings of the National Academy of Sciences, 109(25), 10059-10064.

**An MOI ~200 was used in the attachment assay.**

[3] Kondratowicz, A. S., Hunt, C. L., Davey, R. A., Cherry, S., & Maury, W. J. (2013). AMP-activated protein kinase is required for the macropinocytic internalization of ebolavirus. Journal of virology, 87(2), 746-755.

**An MOI of 200 MOI was used in the internalization assay.**
